# Correlates of HIV seropositivity in young West and Central African women: A pooled analysis of 17 Demographic and Health Surveys

**DOI:** 10.7189/jogh.11.13005

**Published:** 2021-08-10

**Authors:** Christian Bommer, Sebastian Vollmer, Noël Marie Zagre

**Affiliations:** 1 Centre for Modern Indian Studies, University of Göttingen, Göttingen, Germany; 2 Heidelberg Institute of Global Health, University of Heidelberg, Heidelberg, Germany; 3 UNICEF Area Representative for Gabon and São Tomé and Príncipe and to the ECCAS, Libreville, Gabon

## Abstract

**Background:**

Young women in West and Central Africa have been described by the United Nations as being especially vulnerable to HIV/AIDS. Despite a consensus that increased efforts are necessary to address the needs of this particular demographic, correlates of HIV seropositivity in young West and Central African women have not been systematically described. This study fills this gap using a rich set of publicly available survey data.

**Methods:**

For this cross-sectional study, we combined HIV test results for young women (age 15-24 years) with information on demographic, cultural and socioeconomic correlates from 17 recent Demographic and Health Surveys (DHS) to estimate odds ratios (OR) from fixed effects logistic regression models accounting for potential individual, household-level and contextual risk factors of HIV seropositivity.

**Results:**

The prevalence of HIV seropositivity among young women is higher than for men of the same age in all included surveys, except for the Burkina Faso DHS. An important correlate of HIV seropositivity in young women is early sexual activity (OR = 1.510; 95% confidence interval (CI) = 1.100, 2.072), while higher education is associated with reduced odds of being HIV positive (OR = 0.215; 95% CI = 0.057, 0.820). No significant correlation has been found for individual HIV awareness, but HIV stigma is negatively associated with HIV seropositivity (OR = 0.495; 95% CI = 0.247, 0.990, in the fully adjusted model).

**Conclusions:**

The results demonstrate the need to design effective policies addressing behavioral risks in young women. In particular, increasing HIV awareness alone is likely to be insufficient. Instead, information campaigns need to focus on transforming awareness into behavioral change. Moreover, fostering formal education may be an effective tool in the fight against HIV/AIDS.

Despite several decades of dedicated research and strong policy commitment, HIV/AIDS remains a major health care challenge in many parts of sub-Saharan Africa, with an estimated 1.2 million newly-infected individuals in the region in 2017 [1]. While much attention from policy makers and international donors has been devoted to the Southern and Eastern parts of the continent, where overall HIV prevalence tends to be highest [1], the policy response in West and Central Africa has been deemed insufficient by multiple United Nations agencies, warning about high rates of infections among children and adolescents and a lack of access to testing and treatment [2]. A particular reason for concern is the situation of adolescent girls and young women in the region, who have been described by the United Nations as being particularly vulnerable due to gender-based norms and discrimination, as reflected by reduced access to education and HIV-related information and lower bargaining power in sexual relationships compared to boys [3]. Importantly, based on 2017 UNAIDS estimates, young women in the region represented approximately three out of five HIV/AIDS cases in the age group 15 to 24 years and 58% of AIDS-related deaths in the age group 20 to 24 years [3].

Given these alarming statistics, it is crucial to thoroughly understand the various correlates associated with the elevated HIV risk among young West and Central African women in order to design effective HIV initiatives tailored to local needs. While several previous studies have identified potential risk factors for women, such as early sexual activity [4] or socioeconomic status [5], they have been largely limited to individual or small groups of countries, with the consequence that the validity of findings for the wider regional context remains unclear. At the other extreme are a number of studies that pool multiple nationally representative surveys from West and Central Africa together with data from East and Southern Africa [6-11], making it difficult to derive substantive statements for any single region of interest.

To address these limitations, the present study exploits a rich data set of recent Demographic and Health Surveys (DHS) from 17 West and Central African countries to systematically investigate a large set of correlates of HIV seropositivity in young women. In doing so, established modelling techniques are used that allow for a distinction of individual-level and contextual variables. The study thereby contributes to the literature by highlighting for an especially vulnerable demographic potential key risk factors that have not previously undergone comparative assessment in the context of this high-priority region.

## METHODS

### Data sources

The data used in the present analysis were obtained from the DHS program, a publicly accessible database of cross-sectional surveys administered by ICF International in a large number of low- and middle-income countries [12]. All surveys are nationally representative for women of reproductive age (15-49 years) and contain detailed information on many demographic and health-related indicators. From the database, we extracted for each country the most recent survey wave (published until December 15, 2019) that included information on HIV status and limited the sample to women younger than 25 years of age, following the United Nations’ definition of young women [13].

### Outcome measure

HIV blood testing was performed following a standardized protocol, ensuring comparability and confidentiality of results [14]. The outcome variable for the present analysis was constructed as a binary indicator equal to one if DHS indicated a positive test result and equal to zero in case of a negative result. Indeterminant/inconclusive results as well as all patients who denied consent were classified as missing values.

### Statistical analysis

We estimated a series of increasingly restrictive fixed-effects logistic regression models in order to evaluate the association of individual, household and contextual variables with individual HIV seropositivity. In the most liberal specification, we adjusted for survey-level fixed effects to guard against confounding at the country- or survey-level. In a second step, we further included fixed effects at the level of subnational regions. While this removed the possibility to evaluate regional contextual variables, the inclusion of more restrictive fixed effects allowed us to further relax modeling assumptions. In a next step, we then re-estimated both specifications limiting the analysis to young women who have already heard of HIV/AIDS in order to further assess the role of HIV awareness and stigma, leaving us with a total of four regression models. In contrast to our strategy, several previous studies assessing correlates of HIV seropositivity focused on multi-level modelling rather than fixed-effect approaches [15]. While these models bring the advantage that unobserved heterogeneity at higher levels (eg, region or country) can be explicitly modelled, they have received criticism for invoking hard-to-justify assumptions [16]. The present analysis therefore focused on the described fixed-effect models as preferred approach.

In order to allow for meaningful interpretation of pooled survey results, standard DHS weights were rescaled such that they summed up to the 2018 female population of each country according to World Bank data [17]. Moreover, standard errors were clustered at the primary sampling unit and stratum level, taking into account the sampling approach of DHS. Regression coefficients were transformed to odds ratios (OR). All *t* tests were two-sided and tests with type I errors of less than 10% were considered statistically significant.

### Model covariates

All regression models accounted for sociodemographic characteristics, which have been shown to be associated with HIV status in previous analyses [8,10]. This included respondent age, highest educational level, religious background as well as their place of residence (urban vs rural) and household wealth quintiles. The latter variable was calculated by DHS staff on the basis of multiple household assets using principal component analysis and reflected survey-specific socioeconomic status [18].

Respondents’ attitudes towards HIV were assessed using a set of binary indicators that were summarized with two index variables representing the fraction of responses reflecting HIV awareness or stigma, respectively (see **Table 1** for a detailed description of indicators). Note that in contrast to previous analyses [10], we chose to not construct indices on the basis of principal component analysis in order to avoid losing too much information and to facilitate an intuitive interpretation of results. To make sure that we did not capture a spurious relationship driven by the fact that HIV awareness/stigma may have been higher or lower in individuals who previously learned about their HIV status, we adjusted for previous HIV testing reported by the respondents.

**Table 1:** Indicators for HIV awareness and stigma*

Indicator	Coding
***HIV awareness***	***Fraction of variables coded 1 from list below***
Always using condoms during sex reduces HIV risk	1 if respondent answered **yes**; don’t know counted as 0.
Only having one sex partner reduces HIV risk	1 if respondent answered **yes**; don’t know counted as 0.
Mosquito bites can lead to HIV transmission	1 if respondent answered **no**; don’t know counted as 0.
Sharing food with person who has AIDS can lead to transmission	1 if respondent answered **no**; don’t know counted as 0.
Healthy-looking person can be HIV+	1 if respondent answered **yes**; don’t know counted as 0.
HIV can be transmitted to child during pregnancy	1 if respondent answered **yes**; don’t know counted as 0.
HIV can be transmitted to child during delivery	1 if respondent answered **yes**; don’t know counted as 0.
HIV can be transmitted to child through breastfeeding	1 if respondent answered **yes**; don’t know counted as 0.
Knows a place to get HIV test	1 if respondent answered **yes**.
HIV can be transmitted through witchcraft	1 if respondent answered **no**; don’t know counted as 0.
***HIV stigma***	***Fraction of variables coded 1 from list below***
Would want HIV infection in family to remain secret	1 if respondent answered **yes**; don’t know counted as 0.
Would care for a relative in the household who has AIDS	1 if respondent answered **no**; don’t know counted as 0.
HIV+ female teacher who is not sick should be allowed to teach	1 if respondent answered **no**; don’t know counted as 0.
Would buy vegetables from vendor with AIDS	1 if respondent answered **no**; don’t know counted as 0.

*Table lists questions related to HIV awareness and stigma used for index construction. Each index is defined as the share of responses coded as 1.

Moreover, we accounted for marriage status, distinguishing between polygamous and monogamous marriages, as we hypothesized that women living in polygamous relationships would have less agency and be exposed to greater HIV transmission risk given that their spouses were having sexual relationships with multiple women. In addition, we introduced the age and sex of household heads into the models. While living in a female-headed household might be protective by providing young women with agency, it is also possible that female household heads are widows who lost their husbands to AIDS, generating a positive association to HIV seropositivity on the household level [10]. To guard against this risk, we distinguished between female-headed households in which no adult male (21 years or older) was present and those in which there was.

Sexual behavior and knowledge was measured by four variables capturing whether the respondent had sex before the age of 16, whether she had multiple sexual partners during the 12 months before the interview, whether she used a condom during the last sexual encounter within these 12 months and whether she believes that women are justified to ask their partners to use a condom if he has a sexually transmitted infection. In addition to measuring young women’s understanding that condoms prevent the transmission of sexually transmitted infections, the latter variable represents a further indicator of female agency capturing their willingness to demand appropriate protection from sexual partners. Age at first sex was imputed by age at first cohabitation for women who reported that their first sexual activity happened at first union. Finally, we accounted for media consumption by creating a binary indicator for whether respondents watched television at least once per week, as media consumption could potentially influence health-related behavior and attitudes.

It should be noted that questions on television viewing habits and women’s opinion on asking partners to use a condom were not included in the Congolese questionnaire. Similarly, religion was not inquired in the DHS for Niger. Rather than entirely removing these countries from the analysis, we created separate categories “not surveyed” for these cases. Finally, Guinea was excluded for regression models using HIV stigma as most stigma-related questions were unavailable in the Guinea DHS.

To construct contextual variables, we calculated regional-level averages for a subset of the described variables, allowing us to capture network effects with respect to socioeconomic status and HIV attitudes. In order to estimate the influence of local economic activity, we further introduced an innovative satellite imagery-based measure for the average night time light emissions within respondents’ region of residence in the year of survey start [19]. For surveys starting after 2013, satellite data was unavailable, and we used 2013 night time light emissions instead. Similar types of measures have been widely applied within development research and have been previously shown to be a good proxy for economic activity where GDP data are unavailable [20].

### Ethics

This retrospective study was exempt from seeking ethical approval, as only publicly available, anonymized secondary data were used. Procedures and questionnaires of the Demographic and Health Surveys used in this study have been reviewed and approved by the ICF Institutional Review Board. All participants in the Demographic and Health Surveys gave informed consent before taking part in the surveys.

## RESULTS

**Figure 1:**
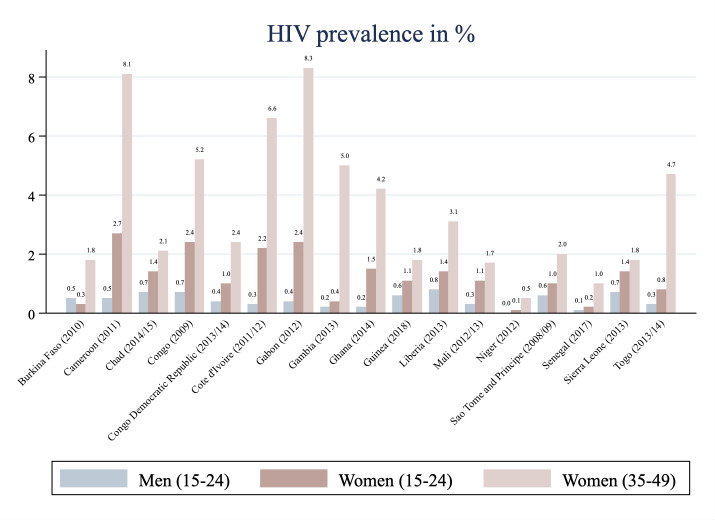
HIV prevalence in young women relative to same-age men and older women. Source: Figure created by authors based on DHS program’s STATcompiler (https://www.statcompiler.com/en/).

We depict summary statistics of HIV seropositivity per included country in **Figure 1**. Surveys were conducted between 2008 and 2018. Prevalence of HIV seropositivity in young West and Central African women (15 to 24 years) ranges from only 0.1% (Niger) to 2.7% (Cameroon). For comparison purposes, we report prevalence estimates for other demographic groups included in the selected surveys. While figures for young women tend to be lower than those for women in the age group 35 to 49 years, they exceed HIV prevalence among 15 to 24 years old men in all but one country (Burkina Faso). The difference is particularly pronounced in Cameroon where HIV prevalence for young women exceeds that of young men by 2.2 percentage points.

**Figure 2:**
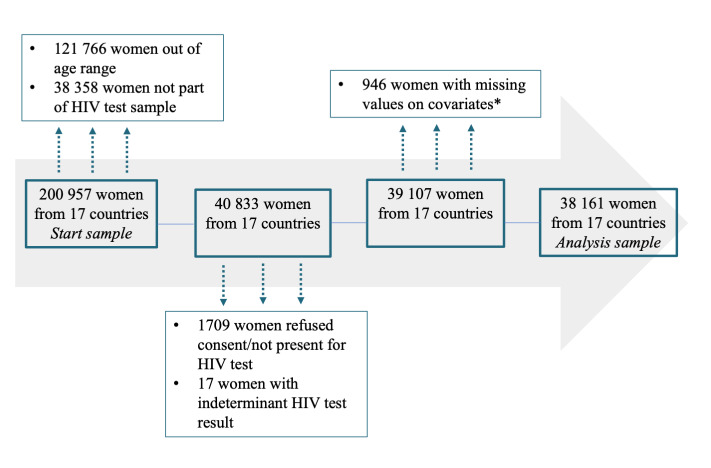
Sample selection. The asterisk indicates that the reported number of women excluded from the analysis due to missing values on covariates does not include women who were not surveyed with respect to religion, TV viewing habits and opinion on asking partner for condom use if he suffers from a sexually transmitted infection.

To investigate whether HIV seropositivity among young women is associated with sexual activity, marriage status and other potential determinants, we next estimate multivariate regression models as described in the methods section. **Figure 2** provides a detailed overview of the selection procedure for the sample constituting the basis for the main analysis. From the initial sample (pooling all 17 surveys) of 200 957 women in the age range 15 to 49 years 121 766 were excluded because they were older than 24 years and 38 358 because they were not part of the households selected for HIV testing. A further 1726 observations were not included because respondents refused to be tested or because HIV tests yielded inconclusive results. Finally, for 946 women, data on important covariates were missing, leaving us with a final analysis sample of 38 161 women from 17 countries.

We present regression results for the analysis sample in **Table 2**. In the following, all results are reported in terms of odds ratios (OR) along with 95%-confidence intervals (95% CI). As described in the methods section, we distinguish between four models. The first specification (column 1) is adjusted for a large set of individual, household and contextual variables but does not contain detailed information on HIV awareness and stigma. Accordingly, age is positively associated with HIV status, whereby the odds of HIV seropositivity are 53% higher among women aged 20-24 years compared to those aged 15-19 years (OR = 1.527, 95% CI = 1.033, 2.259). By contrast, heaving heard of HIV is not significantly associated with HIV seropositivity.

**Table 2:** Regression results: correlates of HIV seropositivity*

	(1)	(2)	(3)	(4)
	**HIV+**	**HIV+**	**HIV+**	**HIV+**
**Age (in years, base: 15-19):**				
20-24	1.527‡ (1.033, 2.259)	1.558‡ (1.075, 2.257)	1.556‡ (1.018, 2.377)	1.615‡ (1.093, 2.385)
**HIV knowledge and attitudes:**				
Ever heard of AIDS	1.568 (0.767, 3.208)	1.490 (0.746, 2.977)		
Ever tested for HIV	1.890§ (1.361, 2.624)	1.815§ (1.330, 2.478)	1.892§ (1.350, 2.653)	1.779§ (1.279, 2.474)
HIV awareness score			0.913 (0.316, 2.636)	0.806 (0.293, 2.220)
HIV stigma score			0.519† (0.265, 1.016)	0.495‡ (0.247, 0.990)
**Marriage (joint base: currently in monog. marriage):**				
Never married	0.563‡ (0.333, 0.952)	0.581† (0.333, 1.014)	0.554‡ (0.316, 0.973)	0.573† (0.319, 1.028)
Polygamous marriage	0.920 (0.472, 1.793)	0.929 (0.490, 1.761)	0.956 (0.463, 1.974)	0.945 (0.478, 1.869)
Formerly married	1.135 (0.615, 2.094)	1.160 (0.572, 2.355)	0.979 (0.504, 1.903)	1.020 (0.482, 2.157)
**Sexual behavior and knowledge:**				
Sex before age 16	1.510‡ (1.100, 2.072)	1.499§ (1.104, 2.034)	1.553§ (1.118, 2.157)	1.552§ (1.126, 2.140)
Multiple sexual partners past 12 mo	1.119 (0.696, 1.800)	1.121 (0.730, 1.721)	1.204 (0.741, 1.956)	1.205 (0.768, 1.891)
Did not use condom with most recent partner	1.057 (0.718, 1.556)	1.037 (0.691, 1.558)	1.059 (0.706, 1.588)	1.044 (0.682, 1.599)
Justified to ask for condom if partner has STI^‖^	1.480‡ (1.034, 2.119)	1.644§ (1.155, 2.341)	1.589‡ (1.077, 2.344)	1.806§ (1.233, 2.644)
**Education (joint base: no education):**				
Primary education	1.424† (0.943, 2.151)	1.352 (0.890, 2.053)	1.359 (0.857, 2.155)	1.278 (0.810, 2.016)
Secondary education	0.968 (0.570, 1.647)	0.924 (0.549, 1.554)	0.922 (0.477, 1.782)	0.867 (0.470, 1.600)
Higher education	0.215‡ (0.057, 0.820)	0.220‡ (0.058, 0.834)	0.202‡ (0.049, 0.826)	0.210‡ (0.052, 0.848)
**Media exposure‖**				
TV at least once per week	0.746 (0.486, 1.144)	0.703 (0.460, 1.074)	0.702 (0.447, 1.103)	0.684† (0.438, 1.067)
**Religion (joint base: no religion)‖**				
Muslim	1.641 (0.533, 5.052)	1.634 (0.552, 4.839)	1.634 (0.499, 5.351)	1.629 (0.518, 5.122)
Christian	2.456 (0.799, 7.546)	2.191 (0.736, 6.528)	2.406 (0.737, 7.859)	2.146 (0.676, 6.811)
Traditional/other	0.850 (0.189, 3.831)	0.770 (0.177, 3.351)	0.982 (0.206, 4.684)	0.823 (0.181, 3.752)
**Sex of household head (joint base: male HH head)**				
Female HH head (no adult male in HH)	1.562‡ (1.057, 2.308)	1.511‡ (1.054, 2.164)	1.513† (0.985, 2.324)	1.431† (0.975, 2.101)
Female HH head (adult male in HH)	1.062 (0.680, 1.658)	0.995 (0.628, 1.576)	1.098 (0.689, 1.750)	1.047 (0.649, 1.687)
**Age of HH head:**				
Age in years	1.010† (0.999, 1.020)	1.012‡ (1.003, 1.022)	1.009 (0.998, 1.020)	1.011‡ (1.001, 1.022)
**HH location**				
Urban	0.757 (0.359, 1.598)	0.802 (0.404, 1.593)	0.614 (0.287, 1.311)	0.672 (0.333, 1.358)
**Wealth quintile (joint base: 1st quintile):**				
2nd wealth quintile	1.492 (0.878, 2.536)	1.390 (0.816, 2.367)	1.321 (0.748, 2.333)	1.230 (0.697, 2.170)
3rd wealth quintile	2.681§ (1.390, 5.170)	2.577§ (1.378, 4.818)	2.639§ (1.273, 5.474)	2.491§ (1.262, 4.916)
4th wealth quintile	5.059§ (2.020, 12.669)	5.110§ (2.026, 12.886)	5.267§ (1.949, 14.230)	5.187§ (1.925, 13.980)
5th wealth quintile	4.838§ (2.045, 11.444)	4.627§ (1.982, 10.800)	4.970§ (1.972, 12.522)	4.671§ (1.888, 11.556)
**Regional contextual variables:**				
Share 1st quintile	0.542 (0.144, 2.041)		0.373 (0.098, 1.421)	
Mean number of school years	0.976 (0.803, 1.187)		1.113 (0.863, 1.436)	
Mean night time light emissions	1.000 (0.977, 1.023)		0.993 (0.973, 1.013)	
Share heard of HIV	0.511 (0.046, 5.615)			
Mean HIV awareness score			0.001§ (0.000, 0.125)	
Mean HIV stigma score			0.014 (0.000, 68.016)	
**Fixed effects:**				
Survey	×		×	
Subnational region		×		×
**N**	38 161	29 167	33 376	25 373

HH – household, STI – sexually transmitted infection

*Table shows odds ratios with 95% confidence intervals in brackets. Models using region fixed effects (columns 2 and 4) have lower number of observations as regions without variation in HIV status were excluded from estimation. Columns 3 and 4 do not include data from Guinea, as most questions on HIV stigma were not available in the respective survey.

†Significance levels of 10%.

‡Significance levels of 5%.

§Significance levels of 1%.

‖Category “not surveyed” not shown.

Moreover, early sexual activity, compared to sexual activity starting from age 16 or later, is associated with 51% higher odds of HIV seropositivity (OR = 1.510, 95% CI = 1.100, 2.072). Similarly, we find evidence that never having married is associated with reduced odds of HIV seropositivity (OR = 0.563, 95% CI = 0.333, 0.952), although results do not indicate that polygamy is a statistically significant correlate. In contrast to what was hypothesized, respondents who believe women are justified to ask for condom use if their husbands have a sexually transmitted disease face elevated odds of HIV seropositivity (OR = 1.480, 95% CI = 1.034, 2.119), while both having had multiple sexual partners and not having used a condom at the most recent sexual encounter during the past 12 months turn out to be statistically insignificant.

Turning to social and cultural factors, it can be observed that having primary education relative to no education is weakly associated with 42% higher odds of HIV seropositivity (OR = 1.424, 95% CI = 0.943, 2.151), while higher education (OR = 0.215, 95% CI = 0.057, 0.820) but not secondary education is correlated with a reduction in odds. Moreover, we do not find statistically significant evidence that regular television watching or various religious affiliations are associated with the odds of being HIV positive. The results further show that young women living in female-headed homes face significantly higher odds than those in male-headed households (OR = 1.562, 95% CI = 1.057, 2.308) only if no adult male is present. Moreover, large and highly significant odds ratios can be observed for high household wealth quintiles (relative to the poorest quintile), while we find no significant association for the urban vs rural location of households. Among the set of regional variables, neither the fraction of households from the poorest quintile nor average school years turn out to be statistically significant. Similarly, local economic activity, proxied by the average night time light emissions within respondents’ region of residence, is not significantly correlated with HIV status. Finally, we find no statistically significant evidence that the share of respondents who heard of HIV is associated with HIV seropositivity.

We assess the robustness of key modelling decisions in the remaining columns 2 to 4. Accordingly, when replacing the contextual variables by regional fixed effects (column 2), results for all individual and household-level variables remain highly persistent, with the exception that the association for primary schooling turns insignificant. Moreover, they remain very similar when estimating the model for the sub-sample of women who have already heard of HIV and adding HIV awareness and stigma scores, regardless of whether the model contains contextual variables (column 3) or regional fixed effects (column 4). While the mean HIV awareness score within a region is negatively associated with HIV seropositivity (OR = 0.001, 95% CI = 0.000, 0.125), no significant association can be observed for individual HIV awareness scores. Moreover, individual HIV stigma correlates with a reduction in the odds of HIV seropositivity (OR = 0.495 95% CI = 0.247, 0.990 in fully adjusted model).

## DISCUSSION

This study describes key prevalence patterns and correlates of HIV seropositivity in young West and Central African women using a large data set of Demographic and Health Surveys from 17 countries. Survey-level prevalence estimates confirm that young women are typically more often HIV positive than men of the same age, suggesting that they are particularly vulnerable. Based on multivariate analyses accounting for regional and country-level confounding, we find that early sexual activity is a highly robust correlate, which is in line with previous results for women of childbearing age in sub-Saharan Africa [10]. However, we do not find significant evidence that other self-reported risky sexual behavior (multiple sexual partners and lack of condom use during the past 12 months) is associated with increased odds of HIV seropositivity, although we are unable to fully rule out that this could be driven by sample size limitations. Moreover, a striking finding is that respondents who believe women are justified in asking their partner to use a condom if he suffers from a sexually transmitted infection are significantly more likely to be HIV positive than those who do not. A potential explanation could be that these women are aware of their positive HIV status due to a previous test and may have changed attitudes after learning about their HIV status. However, given that we control for past HIV tests to guard against this type of bias, this explanation becomes unlikely. Alternatively, women who answer in the affirmative when being asked such a sensitive empowerment- and sexuality-related question, might be less conservative and more likely to engage in risky sexual behavior in ways not otherwise captured by the model. More research is however needed to confirm this interpretation.

The previously mentioned importance of early sexual activity seems to provide support for abstinence-based information and education campaigns that are widely implemented throughout sub-Saharan Africa. Evidence from Kenya, however, suggests that such campaigns are unlikely to reduce the incidence of unprotected sex [21,22]. Providing teenagers and young adults with information on HIV-related risks in order to influence risky sexual behavior rather than sexual activity itself may therefore be more promising. Nevertheless, our results show that individual HIV awareness is not significantly associated with HIV seropositivity. While this may seem puzzling, the result mirrors previous evidence for childbearing-age women in sub-Saharan Africa.[10] A potential explanation is that HIV awareness alone may not necessarily translate into behavioral change. Indeed, evidence from randomized controlled trials shows that informational and behavioral interventions alone rarely succeed in modifying behavior in a way that effectively reduces HIV transmissions [23]. However, a field experiment in Kenya suggests that teenagers’ behavior is responsive to information on relative risk of HIV infection by partner’s age [21]. Moreover, recent evidence from a television-based intervention in Nigeria demonstrates that effective behavioral change is possible if information is transported in a way that facilitates recipients’ emotional involvement [24]. Finally, group dynamics are likely to be important, as shown by the strong negative association for regional HIV awareness in our model. Interestingly, individual HIV stigma has been found to be negatively associated with HIV seropositivity and comparable results have previously been interpreted as merely capturing overall higher prejudice in low prevalence settings [10]. However, the fact that we control for mean HIV stigma at the region level as well as for regional fixed effects raises the question whether this explanation alone is fully plausible. Instead, while having severe adverse consequences for those who are the targets of HIV-related prejudice [25], HIV stigma itself may be protective for prejudice holders by making them more cautious.

Our results further show that while higher socioeconomic status is associated with increased odds of HIV seropositivity, high levels of formal education correlate with reduced odds, suggesting that a further strengthening of school systems could be a powerful tool in addressing HIV risks in West and Central Africa. Finally, women residing in households with a female household head and no adult male in the household have elevated odds of HIV seropositivity, which could be explained by the fact that these households may be more likely to have lost household members to AIDS and thus face elevated HIV risks [10]. In contrast, the absence of a significant association for residing in a female-headed household where adult males are present suggests that an independent household head effect may not exist.

Several limitations apply to this study. First, the use of observational cross-sectional data implies that the described associations are not necessarily causal. While efforts were made to address endogeneity with the usage of survey and regional fixed effects, the nature of the data makes it impossible to fully control for confounding at the individual or household level. Second, despite a relatively large sample size, it is important to stress that insignificant associations do not constitute proof for the absence of effects, as power issues might have limited our ability to discover statistically significant relationships. For instance, in a related study on HIV determinants in sub-Saharan Africa [10], the authors caution that limited variation in contextual variables prevented them from fully exploring their effects. With that said, we partly circumvent this problem by focusing on regions only rather than including country-level contextual variables – using average regional night time light emissions as an innovative measure for economic activity at the regional level and thus gaining more variation in this dimension. Third, while our analysis covers large parts of West and Central Africa, the unavailability of HIV testing data from Nigeria and some other countries implies that the external validity of results could be slightly reduced. However, given the broad sample of countries studied in the present analysis, the findings still remain highly informative for the region.

## CONCLUSIONS

Taken together, this article highlights important relationships between HIV seropositivity and potential risk factors in young women. In particular, we identify early sexual activity as an important correlate, but we question the effectiveness of abstinence-related education campaigns. Moreover, our results suggest that increasing HIV awareness alone is likely to be insufficient given an overall lack of association between individual HIV awareness and seropositivity. Instead, more involving strategies may be necessary that reach young people not only on an informational level but also emotionally. Finally, fostering formal education may be powerful in further reducing HIV incidence in West and Central Africa.
